# Racial and Social Impacts of Diabetes Mellitus: An Autobiographical Case Report

**DOI:** 10.7759/cureus.20211

**Published:** 2021-12-06

**Authors:** Tyler K Smith

**Affiliations:** 1 Department of Pediatrics, University of Missouri-Kansas City School of Medicine, Kansas City, USA; 2 Department of Pediatrics, Children's Mercy Kansas City, Kansas City, USA

**Keywords:** underrepresented in medicine, minority population, minority health, implicit bias, diversity and equity in medicine, diversity and inclusion, racial and social impact, healthcare disparities, diabetes mellitus, autobiographical case report

## Abstract

Type two diabetes mellitus is a chronic medical condition encountered by physicians providing medical care to adult and pediatric patients. This autobiographical case report discusses type two diabetes from the perspective of positive and negative interactions with the healthcare system in managing diabetes mellitus, especially for a physician of color and underrepresented in medicine. Bias and assumptions occur for some people diagnosed with diabetes mellitus or presumed to have the disease based on age, body habitus, comorbidities, lived environment, race, and ethnicity. I specifically address the social implications of bias experienced by persons of color strictly based on race and ethnicity. Intensified awareness about systemic and institutional racism in healthcare warrants eliminating the inequities and disparities in the medical management and treatment of diabetes mellitus.

## Introduction

Advances in diagnosing, treating, and managing diabetes mellitus have evolved since the first descriptions of the disease by ancient Egyptians over 3000 years ago [1]. Gone are the days of diagnosing diabetes mellitus by the sweet smell and even taste of patients’ urine or managing symptoms with calorie restriction [2]. The discovery of insulin by Canadian surgeon Frederick Banting and continued support for good nutrition and exercise promoted by physicians Reginald Fitz and Elliott Joslin brought the advent for revolutionizing medical treatment and management of diabetes care [2,3]. Additionally, the creation of glucometers and apps for mobile devices expanded the technology for efficiently monitoring blood glucose levels away from the physician’s office. These devices kept people with diabetes healthier longer, out of the hospital, and preventing disease sequelae [2]. Unfortunately, even with improvements to diabetes management and medical care, minoritized and marginalized populations continue suffering from the disease at alarming, disproportionate rates.

Research demonstrates that healthcare disparities and inequities exist for people of color with diabetes mellitus. People identifying as Hispanic/Latinx, Black/African American, Asian, and Native American/Alaskan Native have higher rates of diabetes mellitus compared to their White counterparts [4]. In addition to race and ethnicity, genetics, resource-limited communities lacking healthy food options or walkable spaces, mental illness, access to adequate healthcare and insurance, and obesity are just a few factors contributing to the increased risks for diabetes and comorbid complications [4]. Glycosylated hemoglobin, also known as hemoglobin A1C, serves as a marker for glycemic control, with some people of color having higher values suggesting decreased glycemic control [5]. Limited research demonstrates the cause for persistent racial and ethnic differences in glycemic control; however, some studies suggest an association with social determinants of health, e.g., socioeconomic status, access to quality education and healthcare, as well as neighborhood and community environments where people live, work, learn, play, and interact [5]. Distrust of the healthcare system, including negative encounters with or erroneous, preconceived notions by medical professionals, compounds the hesitancy of some people of color to seek medical care and treatment [6-8].

This autobiographical case report highlights the author’s experiences living with type two diabetes mellitus, including diagnosis, treatment, and the social impacts of the disease. The author candidly shares the challenges of managing diabetes and sometimes the perceived stigma even as a person with a medical background and training. By sharing this account, the author hopes to remind physicians and colleagues to be mindful of their personal biases when providing medical care for patients with diabetes.

## Case presentation

My diagnosis of type two diabetes mellitus ironically occurred while in medical school during my family medicine clinical clerkship. Noon report just finished as my classmates and I prepared for an afternoon of interactive, didactic sessions about diabetes mellitus. The instructor reviewed the disease process and pathophysiology. After the discussion, we proceeded through four learning stations to better understand the experiences of patients with diabetes. These stations included nutritional counseling about a healthy diet such as carbohydrate counting and understanding how to effectively read food labels, subcutaneous injection with normal saline (as comfortable), simulating an insulin injection, performing a fingerstick glucose check, and providing anticipatory guidance to patients about diabetes mellitus. While at the fingerstick station, my blood glucose reading was elevated at over 200 mg/dL. We had just finished having lunch during noon report, so my attending recommended that we recheck my blood glucose at the end of the lecture, which would be more than two hours since I last ate. Without feeling concerned or worried, I continued rotating through the respective educational stations. As the session ended, I rechecked my blood glucose with my attending physician: the level remained above 200 mg/dL after more than two hours postprandial.

Diagnosis

My attending in family medicine officially diagnosed me with type two diabetes mellitus in the clinical office a few weeks after attending the diabetes lecture. My family medical history included diabetes mellitus typically diagnosed in later adulthood and managed through lifestyle changes such as diet, exercise, and sometimes oral medication. Although I experienced thoughts of concern about my new diagnosis, my attending physician-assisted in answering my many questions and providing reasonable recommendations about how to manage my diabetes moving forward. The physician specifically spent time discussing how to prevent nephropathy, neuropathy, retinopathy, and other complications of diabetes through appropriate regulation of blood glucose levels and maintaining hemoglobin A1C levels below 7%. The compassion, patience, and support shown to me by the attending physician not only alleviated some of the angst of my diagnosis but also provided me with the confidence that I could manage my disease. In retrospect, I remember learning that I was at risk for diabetes mellitus prior to starting medical school. I, unfortunately, did not have the same positive experience learning about my potential for diabetes compared to learning about my actual diagnosis.

I went to my primary care clinician for a comprehensive, well adult examination prior to starting medical school. My clinician ordered lab tests for completeness since I would be moving out of state to attend medical school. I thought nothing of the exam or my blood work until I received a telephone call from my clinician’s office stating that I needed to make an appointment to review my lab results. As I was now back home with my family and more than a 12-hour car ride away from my clinician’s office, I confirmed my identity and requested my lab results over the telephone. The person on the other end of the telephone informed me that my results must be given in person, and if I did not come in for an appointment as soon as possible, I was going to die. The person was not referring to that we would all die one day, but that there was something concerning with my test results placing my life in peril. Being in my early twenties at the time, worry set in that I had a terminal or incurable illness with something terrible happening to my body. I mobilized quickly and had an appointment with my primary care clinician within one week. Barely addressing the clinician with my usual pleasantries, I started firing off my questions beginning with if I was dying and what medical attention I needed. My clinician looked perplexed, asking what I was told as to the reason for the appointment. Sharing the “you could die” story, my clinician apologized for the language used to convey information about my lab results, including the unnecessary worry, created. I learned my lab results showed the potential for pre-diabetes. Not being in medical school yet, I had no context of what it meant to have pre-diabetes or the potential implications for my health. Did this mean I have diabetes? Would I have diabetes later, and if later, when?

Physicians and healthcare professionals are trained to care for the person and not the disease through effective communication with patients by acknowledging the power of words and how they matter in patient outcomes. Medical education includes training with standardized and real patients to provide information using patient-centered communication and terminology understandable to patients. Patient-centered communication entails active engagement with patients through listening in order to learn about patients’ perspectives about their health when communicating medical information. Studies demonstrate that patients are more inclined to adhere to prescribed medical treatment and management when directives provided by clinicians use patient-centered communication [9]. Even though healthcare professionals receive training in speaking to patients, some people are better skilled at the technique than others. In addition to healthcare professionals’ abilities in talking to patients is the potential bias interjected into the words and language used during patient encounters. I am uncertain of the intentions of the nurse I spoke to on the telephone about my lab results, but I know the personal impact on me. I felt the nurse was insensitive with their choice of words. I felt the nurse believed that I was not serious and did not care about my health. And for a moment, knowing the nurse had my medical chart, I felt the medical comments made were based on my being a person of color.

Lifestyle changes

My initial treatment for type two diabetes mellitus included lifestyle changes and medication. Exercise became incorporated into my regular routine, such as going to the gym using the elliptical machine, lifting light weights, and taking brisk, 30-minute walks. I actively engaged in meal preparation, eating lean meats and protein, fruits, vegetables, and monitoring my consumption of carbohydrates. I became more in tune with regularly reading the carbohydrate content on food labels as well as the appropriate serving size. Lab testing showed that my diabetes was mild, and I was started on repaglinide 2 mg by mouth before meals and checked my blood glucose levels twice daily. My diagnosing physician selected this older drug class believing it would provide me with the best glycemic control while also respecting my preference for a manageable medication regimen.

Being in healthcare, I am aware of the correct actions to take as a patient; however, knowing and doing are two different things. I was not the perfect patient, and physicians are sometimes known to be the worst patients. I liked the positive feeling after a good workout, but that feeling only happened if I exercised, and my excuses for not doing so ranged from having no time as a resident to being tired. In addition to my exercise challenges, I have a large sweet tooth and crave carbohydrates, including bread and potatoes. Sizzling hot French fries straight from the fryer at McDonald’s are my weakness.

I quickly learned if I wanted to prevent complications from diabetes mellitus and keep my illness under control, I needed to make active, sustainable lifestyle changes. I created a schedule for working out, including 30-minute walks outside during nice weather, parking my car away from store entrances to increase my distance walked, and taking the steps instead of elevators as often as possible in multilevel buildings. I changed my eating habits by consuming my favorite foods in moderation. Instead of a large French fry from McDonald’s, I opted for the small. I selected healthier menu options when eating out at restaurants and fast-food establishments. I monitored my blood glucose levels paying close attention to how certain foods impacted the reading. My body does not like large quantities of fried foods, so that became a treat. My healthy carbohydrates included quinoa and sweet potatoes, which had a limited impact on my blood glucose levels. Most importantly, I stopped emotional eating. For example, I had a great day: I will eat junk food; or alternatively, I had a bad day: I will eat junk food.

In understanding the potential complications from diabetes mellitus, I am hypervigilant about prevention strategies. To prevent injuries to my feet, I do not walk around the house or outside without some type of foot covering such as socks, slippers, or shoes. I love being pampered with a pedicure, but I am mindful to ensure the cleanliness of the establishment, including sanitized pedicure instruments, regular cleaning of the foot basin, and using disposable, plastic jets to prevent the risk of exposure to infections. I regularly check my feet for any bruises or cuts since the healing process for patients with diabetes can be prolonged. I also examine my feet frequently to ensure the appropriate sensation and check for signs of neuropathy.

Medication

Selecting medications for treating diabetes mellitus is individualized to the person. What works for one individual may not work for another. My initial medication regimen included repaglinide 2 mg three times daily 10 minutes prior to meals. This controlled my diabetes for approximately five years until I recognized that I needed a little more support in managing my daily glucose levels, and metformin 500 mg twice daily was added. I remained on this regimen and found improved glucose control with the addition of sitagliptin which allowed discontinuing my short-acting medication, repaglinide. I would stay on the cocktail of metformin and sitagliptin for five-seven years until I was ready to transition to “pregnancy-friendly” medication.

Physician appointments

With having the diagnosis of type two diabetes mellitus, no option exists for me to not visit with a physician on a regular basis. I make sure to have a physical examination with my primary care physician annually. I have managed my diabetes with and without an endocrinologist. In managing with the endocrinologist, I was able to try different medications to determine which one worked well and which medication did not provide the appropriate control. I worked with my primary care physician to manage my diabetes when my medication regimen stayed the same and my blood glucose levels remained well controlled. Annual dilated eye examinations are a must with my ophthalmologist, and I try to schedule the appointments around the same time each year to remember.

It is worth noting that my first endocrinologist shared that I did not “fit the picture” of a person with diabetes. I was not aware that people with diabetes mellitus have a specific look like someone with a pathognomonic, genetic disorder, or syndrome. My perplexity likely showed in my facial expression as my specialist clarified. Other than my family medical history of diabetes mellitus, my specialist would not have thought that I had diabetes let alone type two. Based on my age of late 20s and average-build body habitus, the expectation would have been that I had type one diabetes. I completed specialized testing confirming my diagnosis of type two diabetes, but I had a lingering feeling wondering how other patients with diabetes might be perceived. Were other patients assumed to have the disease based on race and ethnicity, weight, diet, exercise level, or family medical history? Or were assumptions made about patients based on the communities where they resided?

Due to changes in my health insurance while in fellowship training, my diabetes was managed for a few years by my primary care physician; however, I resumed medical care with a new endocrinologist when I became an attending physician. My new endocrinologist understood my busy schedule as a physician with limited availability for medical appointments. They worked collaboratively with me in making appointments around my clinical schedule, developed a manageable medication regimen as well as created accountability for diet and exercise. Both of my endocrinologists treated me with dignity and respect as a person with diabetes without my feeling that my race negatively impacted my medical care.

Impacts of diabetes mellitus

As a patient with diabetes, knowing my body and how it feels with changes in blood glucose levels was imperative to appropriate management. During my moments of experiencing hyperglycemia (blood glucose >250 mg/dL), my symptoms include increased hunger and thirst, headaches, and feeling more tired than usual. With hypoglycemia (blood glucose < 60 mg/dL), I experienced anxiety, increased heart rate, feeling flushed or hot, sweating, shakiness, irritability, and tingling or numbness in my tongue. The goal is to prevent these uncomfortable symptoms. My hyperglycemia is treated with drinking lots of water, while my hypoglycemia is treated with drinking 4 oz of juice or eating a few fruit snacks that I keep available for these moments. In either situation, I recheck blood glucose levels to ensure that the intervention worked. I have not needed any emergency medical care secondary to my diabetes mellitus, but I am mindful to monitor how my body feels.

Diabetes mellitus not only impacts the patient but family members and friends too. Family and friends assist with managing lifestyle changes while also dealing with the ramifications of diabetes mellitus complications, including immobility after a lower extremity amputation and dialysis during kidney failure. My mother took my diagnosis the hardest as she thought about the potential complications, including the impacts on future pregnancies. She shared her motherly love wishing she had the disease instead of me. She made the difficult decision to forgo donating her kidney due to concerns that I might need a kidney one day. I have never been diagnosed with nephropathy or any other kidney disease, but my mother operated using proactive thought processes should the situation arise.

Knowing my desire to have children one day, I created a plan with my endocrinologist early. Endocrinologists recommend that women with diabetes have a hemoglobin A1C of less than 7% prior to conceiving: my target goal was a level less than or equal to 6.5%. When I knew that I was ready to consider trying to get pregnant, I transitioned to “pregnancy-friendly” medication to prepare my body. In addition to taking metformin twice daily, I discontinued sitagliptin and added subcutaneous long-acting insulin, insulin glargine, and as-needed short-acting insulin, insulin aspart, to cover my meals containing extra carbohydrates. As someone who does not like needles, specifically needles piercing my body, I had to get used to the subcutaneous injections, which surprisingly were not terrible. In addition to my regular fingerstick glucose checks, I used a Dexcom (DexCom Inc, San Diego, California) device attached to my abdomen or back to continuously monitor my blood glucose levels. Dexcom provided real-time glucose readings to better understand the causes for my low and high numbers. The device is the same as the one worn by Nick Jonas that crashed the internet: ask an adolescent for more information. While I liked the spirit of the device and the information provided, it created moments of annoyance. Twice daily quality checks were needed for glucose readings, and the transmitter needed to be close to the reading device. Air travel was a nightmare as TSA checkpoints at the airport required a medical note, and avoiding the scanner with a physical pat-down was needed. Since my time using the device, Dexcom has made the monitoring process much easier with the ability to track glucose levels on a mobile device.

In speaking about the impacts of type two diabetes mellitus, I would be remiss if I did not share the assumptions made about people with diabetes. Some people give judging looks when checking blood glucose levels or worse, thinking there are issues with addiction when injecting insulin prior to a meal at a restaurant. Unfortunately, some health professionals and others assume that people with diabetes are mostly persons of color, overweight or obese, non-compliant with taking medication, or lackadaisical about their health. Numerous family members and friends describe experiences of long discussions during medical appointments with a clinician repeatedly asking about prescribed diabetes medications. Surprise follows the interrogation when the physician learns that a person of color only takes a daily multivitamin and as-needed over-the-counter pain relievers. Regrettably, some health professionals assume patients of color tell a falsehood about their medical history and therefore are non-compliant with taking medication. People of color who are older, possibly overweight or obese, admittedly lead a sedentary lifestyle, and reside in a resource-limited community do not all have diabetes. In fact, there is no body habitus that predicts who will have diabetes - maybe risks, but not disease.

## Discussion

I have now been living with diabetes mellitus for more than 15 years. I remain healthy, but I still have my own challenges managing the disease. In efforts to decrease my daily doses of long-acting insulin, I researched methods to naturally decrease my blood glucose levels. I started drinking a homemade tea of fresh or dried sage every morning. Sage naturally decreases blood glucose levels. I learned drinking one cup of sage tea daily sufficiently maintains my blood glucose throughout the day, while two cups creates increased moments of hypoglycemia. From this daily practice, I have decreased my long-acting insulin to under 10 units daily. Patients should consult their physician and/or specialist before embarking on natural treatments and remedies that could impact their diabetes mellitus, including medications and disease management. I share what I continue learning about my type two diabetes in hopes that it will help inspire others, including my patients.

Physicians' training entails not sharing our story with patients as clinical time is about the patient's needs and not the physician's. But being a general pediatrician and caring for an adolescent experiencing difficulties with managing their diabetes, I cannot suppress my journey. I care for the adolescent's non-compliance with taking their medication, not checking glucose levels regularly, over-indulging in the wrong foods, and just being plain tired of being the person with diabetes. I create a safe space and speak the truth to my patients. Diabetes sucks and is not fun to manage, but I still encourage my patients. I share that I am living with diabetes, and my patient can too. I share that I am living out all my dreams, and my patient can too. For some patients, just knowing that someone cares to understand their journey and struggle living with diabetes can make all the difference in treatment and management.

Other than my pre-diabetes experience, I have had fairly positive interactions with my healthcare team in managing my diabetes mellitus, which is untrue for some patients of color. Despite health professionals' training in compassionate and empathetic care as well as quality improvements in health systems for managing diabetes, racial and ethnic disparities continue to persist. Hispanic/Latinx, Black/African American, Asian, and Native American/Alaskan Native patients are less likely to participate in diabetes care management, whether self-directed or performed by a clinician with some Asian identities having worse diabetes management compared to other minoritized populations [10,11]. Reasons for poor management include both clinician and patient influences. Cultural differences, time restrictions, and inadequate staff support are factors impacting clinicians' management of patients with diabetes while distrust of the healthcare team and system, cultural differences, and language barriers impact patients' adherence to diabetes treatment [12]. Research suggests implementing programmatic changes at the health professional and medical institution-level, including cultural competency training for clinicians, expanding healthcare teams through the integration of community health workers and allied health professionals, and providing culturally and linguistically appropriate medical care [11]. The disparities in the treatment and management of diabetes mellitus will continue for people of color until individualized medical care is optimized for all patients.

## Conclusions

Type two diabetes mellitus is a challenging chronic medical condition to manage even for a physician with medical knowledge. Support from family and friends, healthy lifestyle choices including good nutrition and physical activity, disease ownership, and personal advocacy aids in my continued thriving with diabetes. I recognize my privilege of medical knowledge and access to resources for managing my diabetes but acknowledge that other people of color do not experience the same luxury. To continue eliminating the healthcare disparities and inequities for people of color with diabetes mellitus, I implore healthcare professionals to ensure that the medical care they provide to patients is the same care they desire for themselves and their loved ones.
